# Mapping a *Partial Andromonoecy* Locus in *Citrullus lanatus* Using BSA-Seq and GWAS Approaches

**DOI:** 10.3389/fpls.2020.01243

**Published:** 2020-08-19

**Authors:** Encarnación Aguado, Alicia García, Jessica Iglesias-Moya, Jonathan Romero, Todd C. Wehner, María Luisa Gómez-Guillamón, Belén Picó, Ana Garcés-Claver, Cecilia Martínez, Manuel Jamilena

**Affiliations:** ^1^Department of Biology and Geology, Research Centers CIAIMBITAL and CeiA3, University of Almería, Almería, Spain; ^2^Departament of Horticultural Science, North Carolina State University, Raleigh, NC, United States; ^3^Instituto de Hortofruticultura Subtropical y Mediterránea (IHSM-CSIC), Málaga, Spain; ^4^COMAV—Universidad Politécnica de Valencia, Valencia, Spain; ^5^CITA—Universidad de Zaragoza, Zaragoza, Spain

**Keywords:** monoecy, partial andromonoecy, andromonoecy, genome-wide association analysis, bulk segregant analysis sequencing

## Abstract

The sexual expression of watermelon plants is the result of the distribution and occurrence of male, female, bisexual and hermaphrodite flowers on the main and secondary stems. Plants can be monoecious (producing male and female flowers), andromonoecious (producing male and hermaphrodite flowers), or partially andromonoecious (producing male, female, bisexual, and hermaphrodite flowers) within the same plant. Sex determination of individual floral buds and the distribution of the different flower types on the plant, are both controlled by ethylene. A single missense mutation in the ethylene biosynthesis gene *CitACS4*, is able to promote the conversion of female into hermaphrodite flowers, and therefore of monoecy (genotype *MM*) into partial andromonoecy (genotype *Mm*) or andromonoecy (genotype *mm*). We phenotyped and genotyped, for the *M/m* locus, a panel of 207 *C. lanatus* accessions, including five inbreds and hybrids, and found several accessions that were repeatedly phenotyped as PA (partially andromonoecious) in several locations and different years, despite being *MM*. A cosegregation analysis between a SNV in *CitACS4* and the PA phenotype, demonstrated that the occurrence of bisexual and hermaphrodite flowers in a PA line is not dependent on *CitACS4*, but conferred by an unlinked recessive gene which we called *pa*. Two different approaches were performed to map the *pa* gene in the genome of *C. lanatus*: bulk segregant analysis sequencing (BSA-seq) and genome wide association analysis studies (GWAS). The BSA-seq study was performed using two contrasting bulks, the monoecious M-bulk and the partially andromonoecious PA-bulk, each one generated by pooling DNA from 20 F2 plants. For GWAS, 122 accessions from USDA gene bank, already re-sequenced by genotyping by sequencing (GBS), were used. The combination of the two approaches indicates that *pa* maps onto a genomic region expanding across 32.24–36.44 Mb in chromosome 1 of watermelon. Fine mapping narrowed down the *pa* locus to a 867 Kb genomic region containing 101 genes. A number of candidate genes were selected, not only for their function in ethylene biosynthesis and signalling as well as their role in flower development and sex determination, but also by the impact of the SNPs and indels differentially detected in the two sequenced bulks.

## Introduction

Watermelons, genus *Citrullus* (2n=2x=22) Schrad. ex Eckl. et Zeyh., are among the most grown vegetable fruit crops worldwide (Levi et al., 2017), representing a planted area of more than 3 million hectares, and production exceeding 100 million tons annually (http://faostat.fao.org/). The cultivated citron, egusi, and dessert watermelons have been treated as subspecies of the single species *Citrullus lanatus* (Fursa, 1972). Although there are some cross-ability among them, genome organization data suggest to separate them into three different species (Renner et al., 2014; Paris, 2015): *C. lanatus* (Thunb.) Matsum. & Nakai is the dessert watermelon (also known as *C. lanatus* subsp. *vulgaris*); *C. amarus* Schrad. is the citron watermelon (also known as *C. lanatus* subsp. *lanatus*), and *C. mucosospermus* (Fursa) Fursa is the egusi watermelon (also known as *C. lanatus* subsp. *mucosospermus*). The genus is also composed of four other species: the less cultivated *C. colocynth*is (L.) Schrad., (known as colocynth watermelon), and the non-cultivated species *C. ecirrhosus* Cogn*., C. rehmii* De Winter and *C. naudinianus* (Sond.) Hooker f. (Paris, 2015).

Watermelons, like as other members of the family Cucurbitaceae, produce unisexual male or female flowers as well as hermaphrodite or bisexual flowers on the same plant (Poole and Grimball, 1938). According to the manner in which these types of flowers are combined, the flowering pattern of watermelon is either monoecious (M, producing male and female flowers on the same plant), andromonoecious (A, producing male and hermaphrodite flowers on the same plant) or partially andromonoecious (PA, producing male, female, hermaphrodite and bisexual flowers on the same plant) (Rudich and Zamski, 1985; Ji et al., 2015), considering hermaphrodite flowers those that have fully developed stamens with pollen, and bisexual flowers those that show a partial development of stamens and do not produce pollen. The cucurbit fruit is developed from the ovaries of female, bisexual or hermaphrodite flowers, and thus the initiation and the correct development of these flowers are crucial with regard to the level and quality of crop production. Andromonoecy and PA are undesirable traits in watermelon, since bisexual and hermaphrodite flowers need to be emasculated when acting as female parents in the production of hybrid seed (Prothro et al., 2013). They are also undesirable because these traits are usually associated with a reduction in fruit set and fruit quality (Aguado et al., 2018).

In the Cucurbitaceae family, sex expression is controlled by environmental, hormonal and genetic factors. Some changes of sex expression in cucurbits have been attributed to variations in temperature and photoperiod regimes (Manzano et al., 2014; Li et al., 2018). Ethylene is the principal hormone regulating sex expression of cucurbits species. In watermelon, recent studies have demonstrated that ethylene inhibits the transition from male to female flowering and reduces the number of pistillate flowers per plant (Manzano et al., 2014; Zhang et al., 2017).

The combination of three pairs of genes explains the control of sex forms in watermelon: gynoecious (*gy*), andromonoecious (*a*), and trimonoecious (*tm*) (Ji et al., 2015). Andromonoecy has been considered recessive to monoecy, being determined by a single locus with two alleles (*M*, monoecious; *m*, andromonoecious) (Rosa, 1928; Poole and Grimball, 1945; Rudich and Zamski, 1985; Salman-Minkov et al., 2008). Recently, it was demonstrated that the *M/m* locus corresponds to the gene *CitACS4*, which encodes for a flower specific ACS enzyme. This enzyme is involved in the biosynthesis of ethylene, which is required for stamen arrest during the development of female flowers (Boualem et al., 2016; Ji et al., 2016; Manzano et al., 2016). As occurs in the orthologs *CmACS7*, *CsACS2* and *CpACS27A* of melon, cucumber and squash (Knopf and Trebitsh, 2006; Boualem et al., 2008; Boualem et al., 2009; Martínez et al., 2014) a single missense mutation (*m*) in the coding region of *CitACS4*, promotes the conversion of monoecy (*MM*) either into andromonoecy (*mm*), or into PA (*Mm*) (Manzano et al., 2016). The andromonoecious allele (*m*) of *CitACS4* also has some pleiotropic effects on fruit development, for example, shortening and rounding the shape of ovaries and fruits, and reducing fruit set and fruit quality of watermelon (Aguado et al., 2018).

Other ethylene biosynthesis and perception genes are responsible for sex determination in cucurbit species. In cucumber, gynoecy is conferred by the dominant locus *F* (Female), which encodes for an additional copy of *CsACS1* (*CsACS1G*) (Trebitsh et al., 1997; Zhang et al., 2015; Li et al., 2020). The recessive gynoecy of melon is, however, conferred by a loss of expression of the C_2_H_2_ zinc-finger-type transcription factor *CmWIP1*, which directly repress *CmACS7* (Martin et al., 2009). The CRISPR/Cas9 generated mutations of *WIP1* orthologs in cucumber and watermelon also lead to gynoecy, demonstrating that *WIP1* is required for carpel abortion during male flower development in different cucurbit species (Hu et al., 2017; Zhang J. et al., 2019). On the other hand, mutations in the *CsACS11* and *CmACS11* orthologs of cucumber and melon, together with the *CsACO2* of cucumber, completely block female flower development, leading to androecy (Boualem et al., 2015; Chen et al., 2016). This androecious phenotype is the result of the loss of ethylene produced by *ACS11* and *ACO2*, which downregulates the expression of the carpel-aborting gene *WIP1*. The ethylene receptors *ETR1* and *ETR2* also play an important role on sex determination of cucurbits. The ethylene insensitive mutants *etr1a* and *etr2b* of *Cucurbita pepo* both disrupt female flower development (converting monoecy into andromonoecy) and significantly increase the number of male flowers in the plant. This probably indicates that *ETR1* and *ETR2* are able to integrate the two ethylene biosynthesis pathways, perceiving and signaling the ethylene produced by *ACS2/7* as well as that produced by *ACS11* and *ACO2* (García et al., 2020).

We identified some inbred lines that are homozygous for the monoecious allele (*MM*), but exhibited a PA phenotype when grown under high temperature conditions (Manzano et al., 2014). This PA phenotype does not appear to be conferred by the *m* allele of *CitACS4* in heterozygous conditions (*Mm*), but by other allelic or non-allelic gene. The current selection of monoecy by the SNP marker linked to the *M* allele is therefore not enough to prevent the production of bisexual and hermaphrodite flowers. The PA phenotype is similar to that of the previously defined trimonoecious (plants producing male, female and bisexual flowers; Rosa, 1928; Ji et al., 2015), however, we cannot ensure that it is actually the same trait. Previously trimonoecy was stated by the occurrence of the three types of flowers but ignoring the ratio between them. The term PA is therefore more precise since it is defined on the basis of the andromonoecious index (AI), which is an index that score the level of andromonoecy in a plant or accession by considering the ratio between female, bisexual, and hermaphrodite flowers per plant (Martínez et al., 2014).

In order to identify the gene controlling the PA phenotype, we screened for this trait in a large number of watermelon accessions, determining the inheritance of it by means of biparental crossings between monoecious (*MM*) and partial andromonoecious (*MM*) inbred lines. Thereafter, we mapped the trait by using bulk segregant analysis combined with new generation sequencing (BSA-seq), and also by genome-wide association analysis (GWAS). These two methods allow the identifications of DNA markers closely linked to the causal gene for a given phenotype (Giovannoni et al., 1991; Michelmore et al., 1991; Mansfeld and Grumet, 2018). By combining these two approaches, we identified a genomic region on chromosome 1 that regulates the partial andromonoecy trait in watermelon.

## Materials and Methods

### Germplasm and Growing Conditions

Three inbred lines of *Citrullus lanatus* were used in this study: P84 (partially andromonoecious), P86 (monoecious), and P87 (andromonoecious). A total of 47 watermelon accessions coming from two Spanish gene banks: Instituto Universitario de Conservación y Mejora de la Agrodiversidad Valenciana (COMAV) and Centro de Investigación y Tecnología Agroalimentaria de Aragón (BGH-CITA), and 155 watermelon PI accessions taken from the USDA National Plant Germplasm System (NPGS) were also used. All watermelon accessions belonged to the species *Citrullus lanatus* (Thunb.) Matsum. & Nakai. Geographically, 38 were from Africa, 70 from Asia, 76 were from Europe, 10 from North America, seven from South America, and one from Oceania. Two commercial cultivars of *C. lanatus*, Charleston Gray and Calhoun Gray, were also included (**Supplementary Table 1**).

Sex phenotyping of most of the PI accessions was performed in two locations and under two different environmental conditions in 2017 and 2018: open field conditions in Raleigh (North Carolina, United States) in the summer of 2017, and greenhouse conditions in Almería (Spain) in the summer of 2018 (**Supplementary Table 1**). Only the PI accessions showing a stable sex phenotype in each location and each year were used for GWAS analysis. The Spanish accessions were evaluated only during spring-summer 2018 in Almería. In the greenhouse, the plants were grown in 10 L pots under the standard agronomic conditions of Almería.

### Evaluation of Sex Expression Traits and Inheritance of PA Phenotype

The andromonoecious index (AI), defined by Martínez et al. (2014) and adapted for watermelon by Manzano et al. (2016), was used to evaluate the andromonoecy level of each plant, population and accession. Pistillate flowers were scored from 1 to 3 according to their degree of stamen development. Female flowers without stamens were scored as AI=1, while pistillate flowers with medium size stamens were assigned as AI=2 (bisexual flower), and pistillate flowers with complete stamens and pollen were scored as AI=3 (hermaphrodite flowers). Based on these flower scores, the AI of each plant was calculated as the average score for at least 10 flowers of the first 30 nodes along the main stem and lateral branches. The average AI of each accession was calculated from at least 5 plants with a minimum of 10 pistillate flowers evaluated per plant. Plants and genotypes with an AI of between 1 and 1.35 were considered to be monoecious, while those with AI scores between 1.36 and 2.7 were considered partially andromonoecious (PA) and those with AI higher than 2.7 were classified as andromonoecious.

The inheritance of the PA phenotype was determined by formal genetic analysis. The monoecious line P86 was crossed with the partially andromonoecious line P84, and the F1 was self-fertilized to produce F2 and thereafter subsequent selfing generations. From F2 and F3 generations, we selected the plants with extreme phenotypes which were then selfed to produce F3 and F4 offspring, respectively. Parental and offspring generations plants were all phenotyped under greenhouse conditions during the spring-summer season of 2017, 2018 and 2019. The χ^2^ test for goodness-of-fit (*P <0.05*) and homogeneity were used to examine segregation ratios (monoecious: partially andromonoecious) in the F2 and successive selfed generations.

### Genotyping for *M* and *m* Alleles of *CitACS4* Gene

The collection of watermelon accessions were genotyped by real time PCR using Taq-Man probes for the *M* and *m* alleles of the *CitACS4* gene. Individual plants’ leaves were sampled, and DNA extracted using the CTAB method (Murray and Thompson, 1980). The PCR reactions were done with Bioline SensiFAST™ Probe No-ROX Kit, a set of forward and reverse primers amplifying the polymorphic sequence, and two allele-specific probes descriptive of the SNP of interest (C-G). The monoecious *M*-allele probe was labelled with FAM dye and the andromonoecious *m*-allele probe was labelled with HEX reporter dye (metabiom). The BHQ1 quencher molecule was used in both probes (**Supplementary Table 2**). Reactions were performed in the Rotor-Gene Q thermocycler (Qiagen) by using green and yellow channels for FAM and HEX reporter dye, respectively. The annealing temperature in the reaction was 60°C.

In a number of *MM*, *Mm*, or *mm* accessions, a fragment of 1,875 bp covering the complete coding region of the *CitACS4* was amplified by PCR using specific primers designed by Manzano et al. (2016), and sequenced for searching new polymorphisms that could be responsible of the PA phenotype.

### Mapping PA Phenotype with Respect to *CitACS4* Gene

To be able to see whether the PA phenotype is linked to *CitACS4*, we searched for a DNA polymorphism in the gene between P84 (partially andromonoecious) and P86 (monoecious) inbred lines, and analysed the segregation of the phenotype and the genotype in 256 F2 plants. Given that the coding region had no variation between the lines, we amplified and sequenced a 708 bp fragment of the promoter by using the specific primers CitACS4genF6/R6 and F4/R4 (**Supplementary Table 2**). The multiple sequence alignments were performed using Clustal Omega (https://www.ebi.ac.uk/Tools/msa/clustalo/). A single nucleotide variant (Indel) at the position -369 bp respect to the ATG start codon was detected between the two inbred lines.

To study the segregation between the PA phenotype and the *CitACS4* gene, the F2 population of the cross P84XP86 was phenotyped for the AI and some plants having extreme monoecious (AI=1) and PA phenotypes (AI>1.6) were genotyped for the SNV which was detected in the promoter by using Sanger sequencing.

### BSA-Seq Mapping Approach

A total of 256 F2 plants from the cross P84XP86 (2016 assay) were phenotyped for AI. 40 plants having the most extreme phenotypes for both monoecy (plants with AI=1) and partial andromonoecy (plants with AI>1.6) were selected. Two DNA bulks, the monoecious M-bulk and the partial andromonoecious PA-bulk, were constructed by mixing an equal amount of DNA from 20 monoecious and 20 PA F2 plants, respectively. Each bulked DNA was randomly sheared into short fragments of about 350 bp for library construction using the NEBNext^®^ DNA Library Prep Kit. Following end repairing, dA-tailing, and further ligation with NEBNext adapter, the required fragments were briefly PCR enriched with indexed oligos. The qualified DNA libraries were pooled according to their effective concentration as well as the expected data production. Pair-end sequencing was performed on the Illumina^®^ sequencing platform, with the read length of PE150 bp at each end. Raw data obtained from the sequencing contained adapter contamination and low-quality reads. Different quality control steps were used: 1) discard the paired reads when either read contains adapter contamination; 2) discard the paired reads when uncertain nucleotides (N) constitute more than 10% of either read; 3) discard the paired reads when low quality nucleotides (base quality less than 5, Q ≤ 5) constitute more than 50% of either read. The resulting sequencing data was aligned with the reference Charleston Gray genome v1 using BWA (Li and Durbin, 2009) software. The duplicates were removed by SAMTOOLS (Li et al., 2009). Single nucleotide polymorphisms (SNPs) were detected by using GATK HaplotypeCaller (Depristo et al., 2011). ANNOVAR was used to annotate the detected SNPs (Zheng et al., 2019).

BSA-seq analysis was performed by using the R package QTLseqr designed by Mansfeld and Grumet (2018). M- and PA-bulk SNPs were filtered using the function “filterSNPs”, thereby selecting SNPs with: a reference allele frequency between 0.3 and 0.7; total depth between 20 and 100; per sample read depth higher than 10; and genotype quality higher than 99.

Following the method developed by Takagi et al. (2013), the identification of QTLs was based on SNP-index and Δ(SNP-index) parameters, calculated by using the function “runQTLseqAnalysis”. SNP-index is the proportion of reads harbouring the variant that is different from the reference sequence. Δ(SNP-index) was calculated by subtracting the SNP-index of M-bulk from that of the PA-bulk. An average SNP-index of SNPs located in a given genomic interval was calculated using a sliding window analysis, with 1 Mb window size and 10 kb increment. The SNP-index graphs for M-pool and PA-pool, as well as corresponding Δ(SNP-index) graphs, were plotted. The Δ (SNP-index) value should not significantly differ from 0 in a genomic region with no major QTL of the target gene (Takagi et al., 2013). The 95% and 99% confidence intervals of the Δ(SNP-index) were calculated under the null hypothesis of no QTLs—using a simulation analysis of 10,000 replications for each bulk—and plotted alongside the Δ(SNP-index) (Takagi et al., 2013).

### Fine Mapping

The QTLseq data were used to design 11 polymorphic SNP markers distributed equidistantly. The 11 SNPs were converted into KASP markers (Kompetitive Allele Specific PCR, www.lgcgroup.com) and used for genotyping a subset of 220 individuals, following the protocol of LGC Genomics. A genetic map was constructed with the data of the 11 SNP markers using JoinMap^®^ 5 (Kyazma, B.V.). Linkage analysis and marker order were performed with the regression mapping algorithm, and genetic distance was calculated using the Kosambi mapping function (Crow, 1990). We performed QTL analysis using MapQTL6^®^ (Kyazma B.V.) interval mapping analysis.

### Genome-Wide Association (GWAS) Mapping Approach

The genome sequence of ‘Charleston Gray’ and the GBS SNPs are available at the Cucurbit Genomics Database (http://cucurbitgenomics.org; Zheng et al., 2019). A diversity panel of 122 watermelon accessions of *Citrullus lanatus* was used for GWAS. The collection was phenotyped for AI in at least 10 pistillate flower per plant in each accession. Only biallelic SNPs within *C. lanatus* accessions (a total of 12,039 SNPs) were used for GWAS. The analysis was performed with TASSEL software (Bradbury et al., 2007; Gur et al., 2017), using the linear mixed model (MLM), which considers both population structure (Q matrix) and relatedness (kinship matrix), and the generalized linear model (GLM), which does only consider the population structure in the association analysis. Genome-wide significance thresholds of GWAS were determined using the Bonferroni correction at p-value=0.05 (False discovery rate FDR 5%) and p-value=0.01 (FDR 1%) for significant and extremely significant associations, respectively, as described in Li et al. (2012). Principal component analysis (PCA), and Quantile-quantile (Q-Q) plots, where distributions of P values expected the null hypothesis distribution, were performed by using TASSEL software (V 5.2.52) (Bradbury et al., 2007).

## Results

### Sex Phenotyping a Large Germplasm Panel of Watermelon

**Figure 1:**
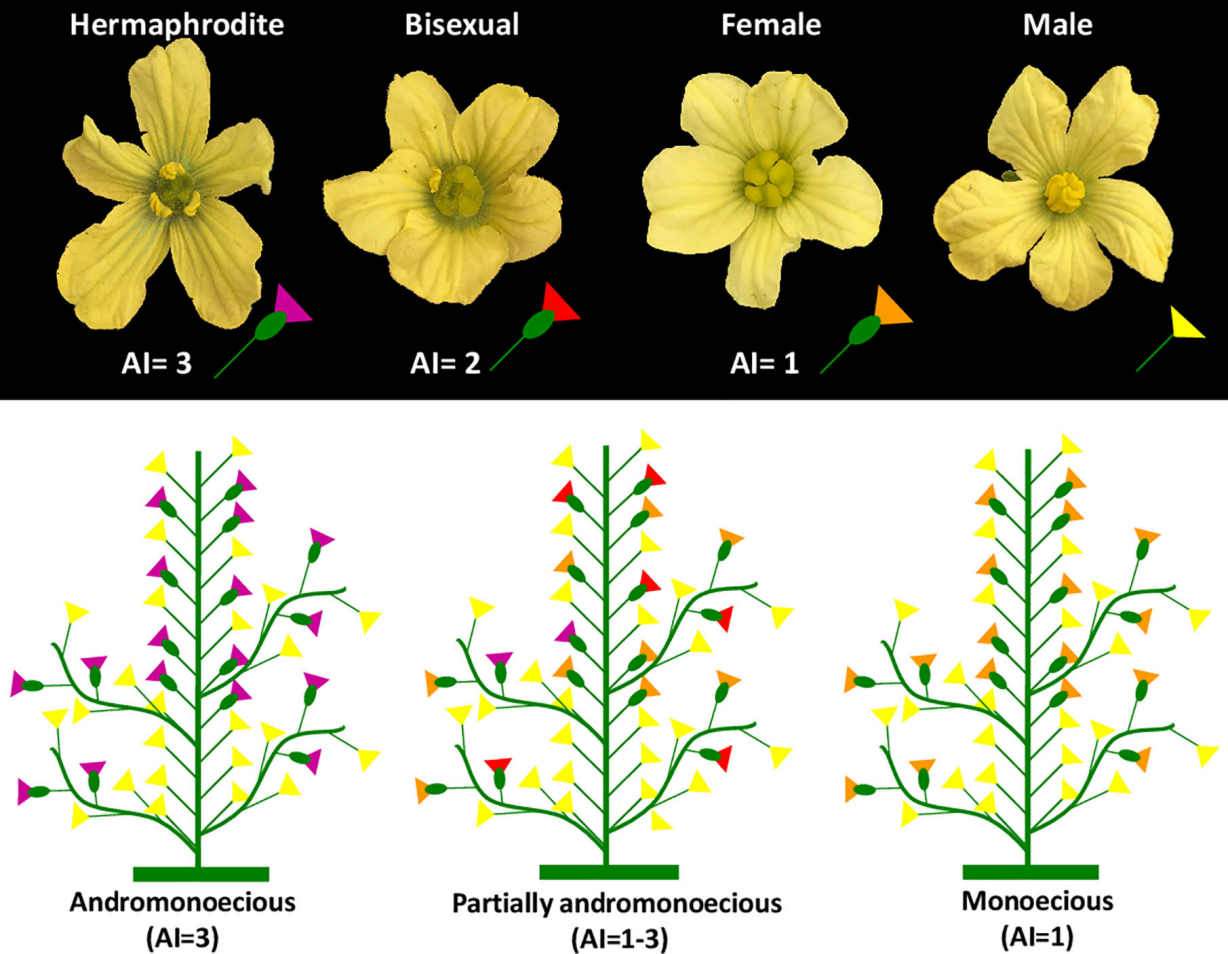
Types of flowers and sex morphotypes in watermelon: monoecious, andromonoecious and partially andromonoecious. Flowers are classified as male (only stamens), female (only carpels), bisexual (producing carpels and some undeveloped stamens) and hermaphrodite (producing both mature carpels and mature stamens with pollen). The Andromonoecious Index (AI) was used to estimate the level of andromonoecy of each plant and accession. For this, female, bisexual and hermaphroditic flowers were assigned the values of AI = 1, 2, and 3, respectively. The AI of each plant was calculated as the average of the AI of a minimum of 10 pistillate flowers per plant. An AI value of 1 corresponds to plants producing only female flowers, and a value of 3 indicates that plants produce only hermaphrodite flowers.

**Figure 1** shows the two main sex morphotypes of watermelon, monoecious (M) and andromonoecious (A), together with the intermediate sex phenotype of watermelon cultivars called partial andromonoecy (PA), the latter being characterized by the production of male, female, bisexual and hermaphrodite flowers on the same plant. We studied the diversity of sex morphotypes among a large germplasm panel of watermelon, comprising 207 watermelon accessions: 155 from USDA-NPGS gene bank, 47 from the Spanish gene banks COMAV and BGH-CITA, plus two commercial cultivars and three inbred lines (**Supplementary Table 1**).

The sex phenotype of each accession was assessed by using the andromonoecious index (AI), using a minimum of 10 pistillate flowers per plant and five plants per accession (**Figure 1**). This index ranges from 1 to 3 and measures the degree of stamen development in pistillate flowers, and therefore the level of andromonoecy of a genotype (**Figure 1**). An AI=1 corresponds to complete monoecy (pistillate flowers are all female), and AI=3 to complete andromonoecy (pistillate flowers are all hermaphrodite). AI between 1 and 3 is assigned to plants and accessions whose pistillate flowers can be either female, bisexual (partial arrest of stamen development) and hermaphrodite (complete development of stamens and pollen) (**Figure 1**). The inbred lines P86, P87, and P84, that were previously shown to have an average AI of 1.02, 3, and 1.55, respectively, were used as control indices of M, A, and PA, respectively.

Taking into account the sex phenotype of these three control genotypes under our environmental conditions, we stablished that accessions with 1≤AI ≤ 1.35 were monoecious, those with 2.7≤AI ≤ 3 were andromonoecious, and those with 1.35<AI<2.7 were partially andromonoecious. In accordance with these criteria, 18% of the phenotyped accessions were classified as monoecious, 43% as andromonoecious, and 39% as partially andromonoecious (**Supplementary Table 1**).

The locus *M/m*, which underlies the ethylene biosynthesis gene *CitACS4*, is the main regulator of monoecy. Homozygous *MM* and *mm* plants are monoecious and andromonoecious respectively, while heterozygous *Mm* are partially andromonoecious (Manzano et al., 2016).

**Figure 2:**
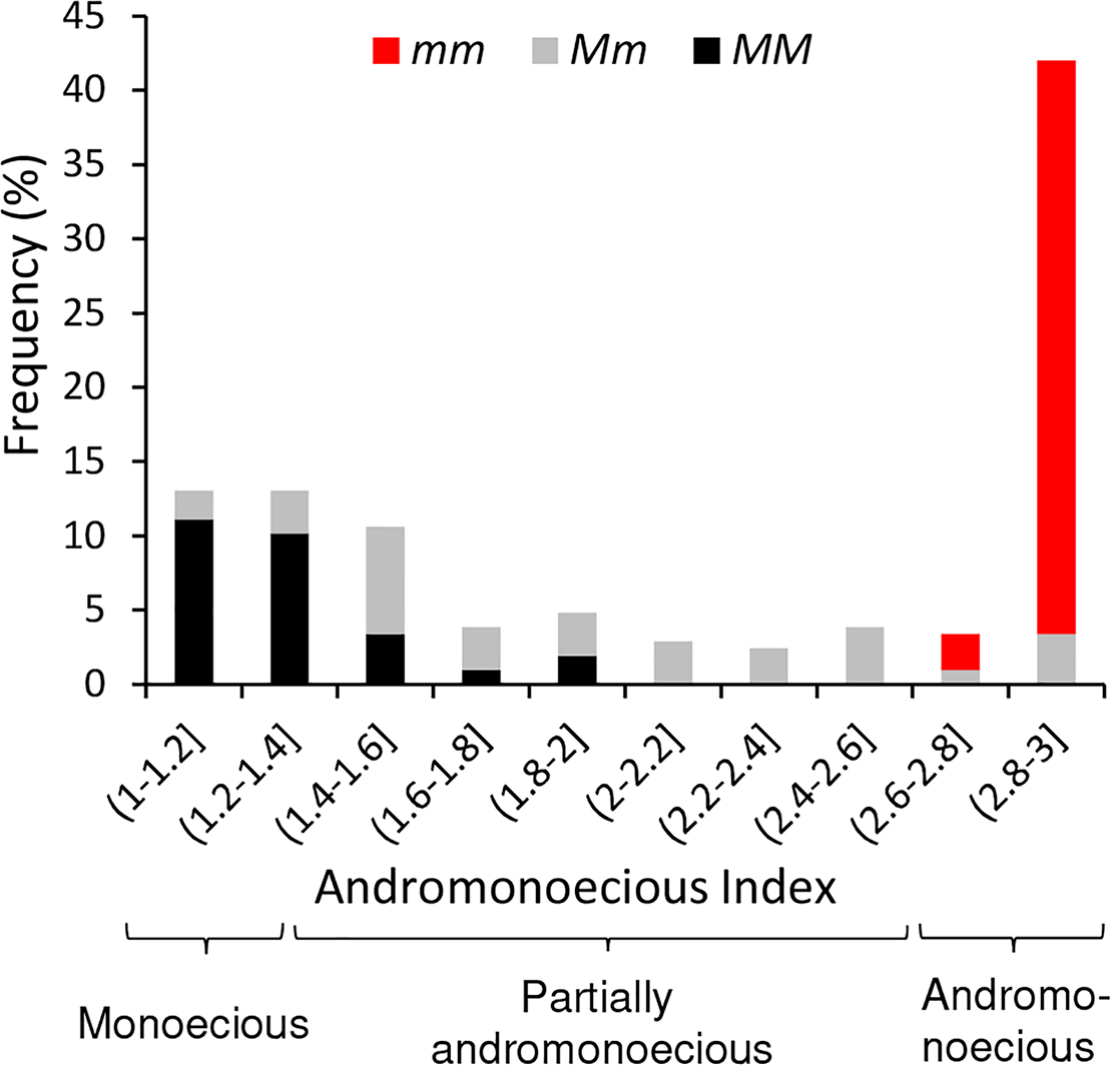
Frequency distribution of Andromonoecious Index (AI) in 207 accessions of watermelon. Bar colors indicate the genotype of accessions for the *M* (*monoecious*) and *m* (*andromonoecious*) alleles of *CitACS4* gene. Given accessions were initially genotyped as a bulk, the *Mm* accessions are those having both *M* and *m* alleles, but plants of those accessions were proved to be *MM*, *Mm*, or *mm*.

So as to investigate whether the variability found in sex determination can be explained solely on the basis of this gene, we genotyped all the accessions for the *M* and *m* alleles of *CitACS4* by using a bulked DNA from five plants of each accession. Some accessions were segregating for the two alleles and exhibited the three sex phenotypes: monoecious, andromonoecious, and partially andromonoecious (**Supplementary Table 1**). All the accessions that were genotyped as *mm* were andromonoecious. Most of the *MM* accessions exhibited a stable monoecy and produced male and female flowers (**Figure 2**). However, a number of accessions were found (BGHZ4849, BGHZ5441, BGHZ5442, BGHZ5993, BGV002674, PI 164992, PI 379237, and Calhoun Gray) that were *MM* but showed a partially andromonoecious phenotype, characterized by the production of male, female, bisexual, and hermaphrodite flowers (**Supplementary Table 1**; **Figure 2**). The PA phenotype of these *MM* accessions could possibly be conferred by an andromonoecious allele on the *CitACS4* gene other than *m*, or by a different gene.

To investigate the occurrence of new andromonoecious alleles in *CitACS4*, the gene was amplified and sequenced in 16 *MM* accessions showing either monoecious or partial andromonoecious phenotypes, along with 4 *mm* andromonoecious accessions (**Table 1**). The control lines P84, P86, and P87 were included in the sequencing analysis. In the coding region of *CitACS4*, we found no variation other than the mutation C to G in the third exon, which is responsible for the andromonoecious phenotype in *mm* plants (Manzano et al., 2016). One single nucleotide variant was found in the first intron, and three in the second intron of the gene, but none of them was linked to the PA phenotype (**Table 1**). Based on this data, we concluded that the partial andromonoecy of *MM* plants is not likely conferred by a *CitACS4* allele. It seems to be caused by a different gene.

**Table 1:** Polymorphic sites of *CitACS4* gene in 20 watermelon accessions.

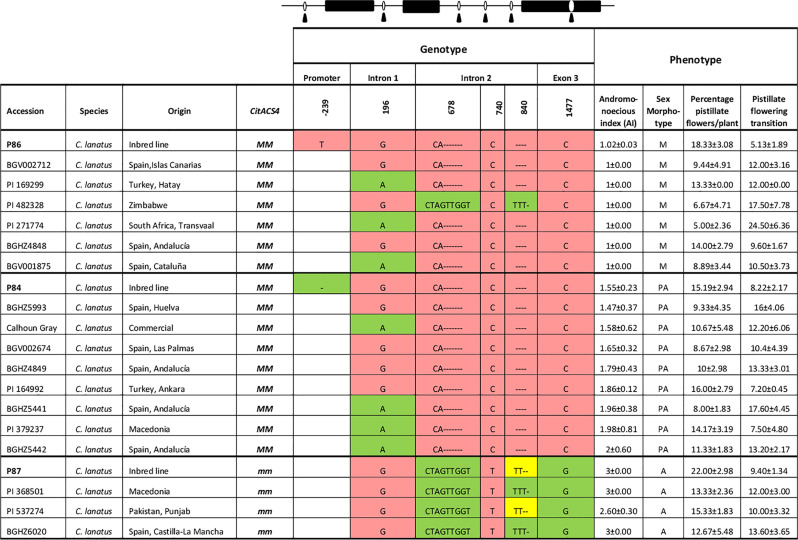

The top shows the structure of the gene, and the identified polymorphic sites. The *M* and *m* alleles are defined by the polymorphism at position 1,477 in the third exon. The sex phenotypes of each accession are also indicated. Pink, green, and yellow boxes indicate the nucleotide variants in the different accessions. The polymorphic site (indel) in the promoter was only analysed in the P84 and P86 parental inbred lines. M, monoecious; A, andromonoecious; PA, partially andromonecious.

### Partial Andromonoecy (PA) Phenotype of P84 Is Conferred by a Single Recessive Gene Other Than *CitACS4*

**Figure 3:**
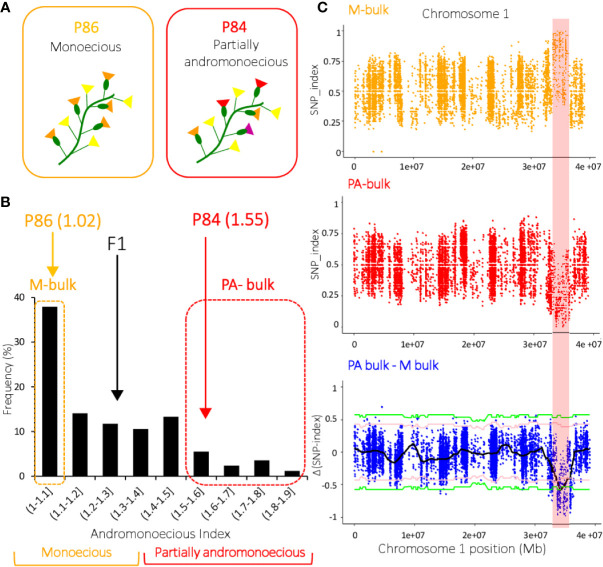
QTL‐seq applied to watermelon F2 progeny identified the locus conferring partial andromonoecy (*pa*) on chromosome 1. **(A)** Phenotype of two watermelon inbred lines P84 and P86 for andromonoecy index. P84 is partially andromonoecious whereas P86 is monoecious. **(B)** Frequency distribution of andromonoecious index in 256 F2 progenies. The average andromonoecy index (AI) for P86 and P84 is indicated. The dashed orange and red boxes indicate the 20 F2 plants with AI=1 that were used to make the M-bulk, and the 20 F2/AI>1.6 plants used to make the PA-bulk. **(C)** SNP‐index plots of M‐bulk and PA‐bulk, and the Δ(SNP‐index) plot on chromosome 1. SNP-index was calculated based on a 1 Mb interval with a 10 kb sliding window. A candidate QTL on chromosome 1 (32.2–36.4 Mb interval) met the criteria that the SNP-index in M-pool was near 1, the SNP-index in PA-pool was near 0, and the Δ (SNP-index) was above the confidence value (P < 0.001). The Δ (SNP-index) plot shows the tricube smoothed Δ(SNP-index) and the statistical confidence intervals under the null hypothesis of no QTL (pink, P < 0.05; green, P < 0.01).

Inheritance of the PA phenotype was studied in the P84 (PA) X P86 (M) cross. P86 is a monoecious *MM* line producing only male and female flowers (average AI= 1.02), while P84 is a partially andromonoecious *MM* line producing male, female, bisexual, and hermaphrodite flowers (average AI= 1.55) (**Table 1**). The average AI of the F1 progeny plants was 1.21, while that of the F2 plants ranged between 1 and 1.9 (**Figure 3**). As stablished above, the plants with 1≤AI ≤ 1.35 were considered monoecious, and those with 1.35<AI<2.7 partially andromonoecious. The F1 was monoecious, and the F2 progeny segregates for 181:75 (M:PA), which fit the expected 3:1 ratio for a single gene (χ^2 =^ 2.52, P value= 0.11; **Table 2**). F3 and F4 progenies of PA plants were always PA, while those of M plants were either M or segregated for M and PA (**Table 2**). This data indicates that the PA phenotype of P84 is conferred by a single recessive gene, the one we have called *pa*.

**Table 2:** Segregation ratio of monoecious and partially andromonoecious in F2, F3, and F4 populations derived from crosses between monoecious and partially andromonoecious inbred lines (P86XP84).

Generation	No. of plants		Expected segregation	χ²	p-value
	Monoecious	Partially andromonoecious			
Parental *P84*		12			
Parental *P86*	12				
F1 (*P84XP86*)	12				
F2 (F1 ⨂)	181	75	3:1	2.52	0.1124
F3					
(*papa* ⨂)		60			
(*PaPa* ⨂)	22				
(*Papa* ⨂)	45	14			
*F4*					
(*papa* ⨂)		50			
(*PaPa* ⨂)	30				
(*Papa* ⨂)	36	13			

The F2, F3 and F4 plants were phenotyped on the basis of their average AI, scored from at least 10 flowers per plant. Monoecious (1≤AI ≤ 1.35) and partially andromonoecious (1.35<AI<2.7). The genotypes for the partial andromonoecious locus (PA/pa) in F3 and F4 generations were assigned on the basis of the segregation ratio. ⨂, self-pollination.

A segregation analysis in the F2 generation of P84XP86 confirmed that *pa* is not an allele of *CitACS4*, but a newly found gene controlling sex determination in *C. lanatus*. The gene *CitACS4*, including exons, introns, and the 708 bp of the promoter, was sequenced in P84 and P86 lines. No nucleotide variation was found in the coding region of *CitACS4* between P84 and P86, but a single nucleotide deletion was detected in the promoter region of P84, at nucleotide position -369 respect to the ATG start codon (**Table 1**). Twenty-five F2 plants were genotyped for this indel, but the deletion did not cosegregated with the PA phenotype in the F2 population of P84XP86. This demonstrated that the PA phenotype of P84 is not conferred by *CitACS4* but by a different, unlinked gene.

### QTL-Seq Identified a Major Locus Controlling PA on Chromosome 1

Bulked-segregant analysis coupled with whole genome sequencing (BSA-seq) was used to map the *pa* locus, which is responsible for the partial andromonoecy of the P84 line. Twenty plants with extreme phenotypes in the F2 from the cross P84 (PA) X P86 (M) were selected and their DNA pooled together to construct two DNA bulks: the M-bulk and the PA-bulk (**Figure 3**). The M and PA parental lines, along with the M- and PA-bulks were resequenced by WGS (Whole Genome Sequencing). Illumina high-throughput sequencing resulted in 55,875,160 short reads (350 bp) from M-bulk (average 18.3X depth and 99.05% coverage), and 55,743,486 short reads (350pb) from PA-bulk (18.11X depth and 99.06% coverage) (**Table 3**).The alignment of the reads to the Charleston Gray v1 reference genome resulted in more than 370,000 SNPs in each of the bulked DNA samples (**Table 3**).

**Table 3:** Summary of sequencing data and alignment result of BSA-Seq.

Sample	Total reads	Mapped reads	Clean data (G)	Q30(%)	% Mapping reads	Average depth (X)	Coverage at least 4X (%)	SNP number
M_bulk	55,875,160	55,202,891	8.40	93.02	98.80	18.30	97.79	370,332
PA_bulk	55,743,486	54,904,988	8.40	92.48	98.50	18.11	97.76	374,678
P84	51,301,618	50,549,756	7.70	92.65	98.53	16.52	97.39	193,904
P86	56,832,990	55,956,004	8.50	92.73	98.46	17.28	97.18	356,384

SNPs from the two bulked datasets were used to run the QTLseqr R package, calculating SNP-index for M- and PA-bulks, and Δ(SNP-index). An average SNP-index was computed in a 1 Mb interval using a 10 kb sliding window, and plotted against the positions along each chromosome of the Charleston Gray genome (**Supplementary Figure 1**). By combining the SNP-index information in M- and PA-bulks, the Δ(SNP-index) was also computed and plotted against the genome positions (**Supplementary Figure 1**). As expected, most of the genomic regions were not relevant to the phenotypic variation (andromonoecious index) and showed identical SNP-index graphs for the M- and PA-bulks. However, a single region on chromosome 1 is the most probable *pa* locus, since it exhibits unequal contributions from P84 and P86 parental genomes (**Figure 3**). The region on chromosome 1 comprised from 32,237,329 to 36,436,269 Mb, had an average SNP-index higher than 0.63 in M-bulk, while the SNP-index in the corresponding region of PA-bulk was lower than 0.33. After examining SNP haplotypes in the M- and PA-bulks, it was found that most haplotypes in the 32.24–36.44 Mb region of chromosome 1 corresponded to P86 (monoecious) in the M-bulk and to P84 (PA) in the PA-bulk (**Figure 3**). The Δ(SNP-index) value should be significantly different from 0 if a genomic region harbors a major QTL of the target gene. At 95% and 99% significance level, only the genomic region on chromosome 1 from 32.24 to 36.44 Mb had an Δ(SNP-index) value that significantly differed from 0 (**Figure 3**). This major QTL on chromosome 1 (QTL1) is therefore most likely to underlie the watermelon PA phenotype.

### Genome-Wide Association Studies (GWAS) Regard to Andromonoecy and Partial Andromonoecy

Genome-wide association studies (GWAS) has proven to be a powerful tool in dissecting the genetic basis of variation for both simple and complex traits. This results in the identification of nucleotide variants that are associated with the trait being sought. Using the nucleotide variation approach, that is, the variation reported in the USDA watermelon collection after genotyping-by-sequencing (GBS) (Wu et al., 2019), we performed a GWAS analysis of the Andromonoecious Index (AI) trait. We filtered out - from all the phenotyped accessions - those traits with no GBS data, and also those showing a high inter-replication variance for the trait in question. Accessions that clearly segregated for monoecy and andromonoecy (accessions with plants having AI=1 or AI=3) were also discarded. A panel of 122 PI accessions was finally selected (**Supplementary Table 1**). A total 15,681 GBS-SNPs with a minor allele frequency of 0.01 or greater and a missing data rate of 5% or less in the panel were used for GWAS. The density of SNPs per chromosome ranged between 1425.54 and 39.55 SNPs per Mb, with an average of one SNP for each 25.3 Kb.

**Figure 4:**
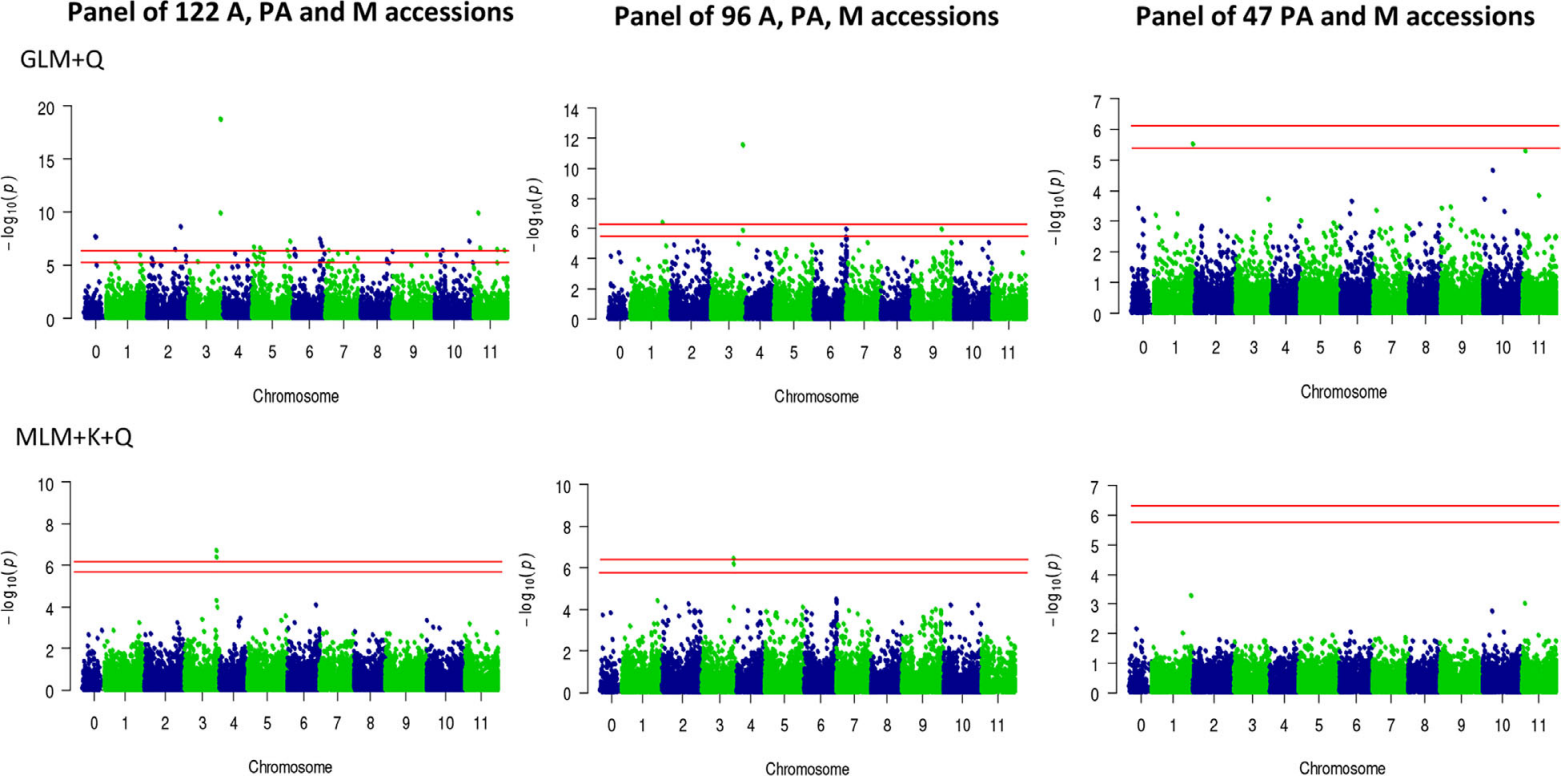
Manhattan plots of GWAS in watermelon for andromonoecious index (AI). Two statistical models have been applied: GLM+Q (General linear model, corrected for population structure), and MLM+K+Q (Mixed linear model, corrected for both population structure and relatedness). The GWAS was performed using populations of differing sizes: 122 accessions, comprising the sex morphotypes A, PA, M; 96 accessions, comprising the morphotypes A, PA and M, and 47 accessions, comprising the morphotypes PA and M accessions. Red horizontal lines indicate the Bonferroni corrected significance thresholds of GWAS at p=0.05 and p=0.01, respectively.

Principal component analysis (PCA) was performed to ensure that any possible associations between SNPs and the trait cannot be attributed to population structure, that is, any kind of relatedness between genotypes in the sample. The PCA plots of accessions panels are shown in **Supplementary Figure 2**. For association mapping, we used both General Linear Model (GLM) and Mixed Linear Model (MLM). The former includes a correction for population structure as covariate (GLM+Q), while the latter also incorporating a correction for kinship (MLM+Q+K), which is used for association mapping. Using the panel of 122 accessions, and using both GLM+Q (p-value = 1.74E-19) and MLM+Q+K (p-value = 1.96 E-7) statistical models, two SNPs at positions 30,740,460 and 30,740,501 on chromosome 3 were found to be significantly associated with monoecy/andromonoecy traits (**Figure 4**). The two SNPs lie approximately 80 kb upstream of the locus *M*/*m* (*CitACS4*), the major causative gene regarding monoecy/andromonoecy in watermelon (Manzano et al., 2016).

Given that the PCA plot for the panel of 122 accessions showed a cluster of andromonoecious accessions that were clearly related, all of them were derived from Africa (**Supplementary Figure 2**), we discarded them and generated a new panel comprising 96 accessions (**Supplementary Table 1** and **Supplementary Figure 2**). In the new panel, GLM+Q resulted in an additional SNP at position 31,376,952 on chromosome 1 that was found to be significantly associated with andromonoecy and PA (p-value = 4.16 E^-7^). Moreover, in an attempt to map the locus controlling partial andromonoecy (*pa*), we also discarded all the andromonoecious accessions (*mm*) of the panel. The remaining 47 accessions (**Supplementary Figure 2**), 17 monoecious and 30 partially andromonoecious, were subjected to the same association analysis (**Supplementary Table 1** and **Figures 2** and **4**). The homogeneous distribution of accessions in the PCA plot with the two first principal component indicated there is no reason to suspect that population structure could influence the GWAS, so as to give false positive associations (**Supplementary Figure 2**). Although the panel size was small, and MLM+Q+K detected no associated SNPs, the generalized linear model (GLM+Q) resulted in only one single SNP of significance on chromosome 1 (position 37,154,162) that was associated with partial andromonoecy (p-value = 2.94E−6). This was situated very close to the locus evidenced by BSA-seq analysis (**Figure 4**).

### Fine Mapping of QTL1

**Figure 5:**
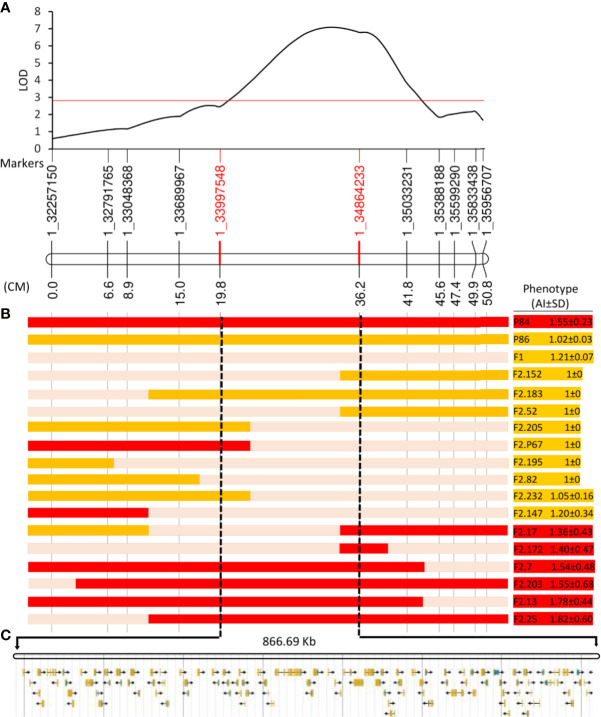
Fine mapping of partial andromonoecy (*pa*) locus. **(A)** Interval mapping result on chromosome 1, showing the logarithm of the odds (LOD) score (y-axis), and the genetic distances for the 11 SNPs (x-axis) that were used. The numbers of the markers correspond to their genomic position in bp. **(B)** Genotypes and Phenotypes of parents, F1, and 15 F2 informative individuals showing recombination in this genomic interval. The color bars show the genotypes along chromosome 1: red, homozygous for P84 allele (partially andromonoecious); orange, homozygous for the P86 allele (monoecious); light pink, heterozygous. The phenotypes for the andromonoecy index (AI) are shown on the right: red; partial andromonoecious (1.35<AI<2.7); orange, monoecious (1≤AI ≤ 1.35). **(C)** The *pa* locus maps on an interval of 866.69 Kb between markers 1_3399758 and 1_34864233, which contains 101 annotated genes.

Since QTL1 has a total size of 4.2 Mb (position 32.24 Mb to 36.44 Mb on chromosome 1), a fine-mapping study was designed to narrow down the genomic interval at the *pa* locus. A total of 220 F2 plants were genotyped for 11 SNPs (identified by the BSA-seq approach) that were about 0.5 Mb distant (**Supplementary Table 3**). Twenty-five informative plants showing recombination events in the region were used to delimit QTL1 within an interval of 867 kb, between markers 1_33997548 and 1_ 34864233 (**Figure 5**). The genotypes and phenotypes of F2 plants (**Figure 5**) clearly indicate that this 867 Kb region cosegregates with the PA trait.

The 867 Kb genomic region contains 101 annotated genes (**Supplementary Table 4**). We investigated whether the function of these genes was related to ethylene biosynthesis and signaling, or with flower development and sex determination (**Table 4**). The narrowest interval contains an F-box gene (ClCG01G020700); it has been reported that some members of this family are known to regulate ethylene response pathway (Qiao et al., 2009), as well as four linked chitinase-like genes (ClCG01G020770, ClCG01G020780, ClCG01G020790, and ClCG01G020800), some of these are known to control developmental processes by regulating ethylene biosynthesis (Zhong et al., 2002; Gu et al., 2019). The 867 Kb genomic region also contains several genes known to be involved in flower development (**Table 4**). The identified genes encode for: an FG-GAP repeat-containing family protein (ClCG01G020030); a lateral root primordium family protein (ClCG01G020040); a Glutaredoxin family protein (GRXs) (ClCG01G020060); GATA transcription factor (ClCG01G020080); a WUSCHEL related homeobox 1 transcription factor (ClCG01G020260); and lastly for an MS5 male sterility family protein (ClCG01G020430) (**Table 4**).

**Table 4:** Annotated genes in the mapping region of the watermelon *pa* locus that are related to ethylene biosynthesis and response, or to flower development.

Gene_ID	Annotation	Function in flower development	References
ClCG01G020030	FG-GAP repeat-containing family protein	Some family members regulate stamen and pollen development.	Zou et al., 2020
ClCG01G020040	Lateral root primordium family protein	The SHI/STY family of transcription factors regulate the development of different plant organs, including stamens and carpels.	Kuusk et al., 2006; Estornell et al., 2018; Singh et al., 2020
ClCG01G020060	Glutaredoxin family protein (GRXs)	ROXY1 and ROXY2 of Arabidopsis are involved in anther development. The double *roxy1 roxy2* mutants does not have anthers and is consequently male sterile.	Reichheld et al., 2010; Xing and Zachgo, 2008
ClCG01G020080	GATA transcription factor	Floral organ development in Arabidopsis. Mutants for the *HANABA TARANU* gene in Arabidopsis have fused sepals and reduced organ number in the four whorls, but especially in petals and stamens.	Zhao et al., 2004; Mara and Irish, 2008
ClCG01G020260	WUSCHEL related homeobox	Lateral organ outgrowth and floral organ fusion in Arabidopsis, Petunia and Medicago.	Matsumoto and Okada, 2001; Yu et al., 2003; Vandenbussche et al., 2009
ClCG01G020430	Male sterility MS5 family protein	Microsporogenesis.	Sanders et al., 1999
ClCG01G020700	F-box family protein	Components of the Ubiquitin-proteosome system for protein degradation. Regulation of EIN2 and EIN3 ethylene signalling elements, and flower developmental genes such as UFO. DDF1-1 of rice control B-class floral homeotic genes.	Chae et al., 2008; Qiao et al., 2009; Duan et al., 2012; Xing et al., 2012; Lin et al., 2016
ClCG01G020770 ClCG01G020780 ClCG01G020790 ClCG01G020800	Chitinase	The Arabidopsis CHITINASE LIKE 1 (CTL1) controls root elongation by negatively regulating ethylene biosynthesis and response. Floral organ development in Arabidopsis and rice.	Gu et al., 2019; Takakura et al., 2000

Searching for candidate genes for the *pa* locus we also investigated the impact of 901 SNPs and 169 INDELs in the mapped region. Forty-one of the SNPs were exonic, and 21 caused non-synonymous changes located in the coding sequences of 17 genes (**Supplementary Table 4**). Of the exonic and non-synonymous SNPs, only two genes—ClCG01G019850 which encodes for a Cytochrome P450, and ClCG01G020620, which encodes for an unknown protein—were found to be distributed in M- and PA-bulk as expected according to the parental phenotypes and the inheritance mode of the trait (**Supplementary Table 4**). Among a total of 169 INDELs, only one was found to be located within a coding region. It corresponds to a frameshift deletion of 38 bp that was found in gene ClCG01G020800, which was annotated in the watermelon genome as a Chitinase-like protein (**Table 4**). The deletion was found solely in the PA parental line (P84) and in the PA-bulk.

## Discussion

### Partial Andromonoecious Phenotype Is Conferred by a Single Recessive Gene Other Than *CitACS4*

As monoecy is an important trait in watermelon, the occurrence of partial or complete andromonoecy not only implies the use of manual emasculation for the production of hybrid seed (Wehner, 2008), but also a reduced fruit set and quality (Aguado et al., 2018). Three watermelon inbred lines, P86, P84, and P87, have been previously identified and characterised; they are representative of monoecy, partially andromonoecy, and andromonoecy respectively (Manzano et al., 2016). The P86 and P87 are very stable lines under different environmental conditions, producing either male and female flowers (P86) and male and hermaphrodite flowers (P87), respectively. The P84 line was more unstable, that is, besides male and female, also developed bisexual and hermaphrodite flowers when plants were grown under a high-temperature regime (Aguado et al., 2018). By taking into account the andromonoecious index (AI) (Martínez et al., 2014), as well as studying the sex phenotypes of these control lines, more than 200 C*. lanatus* accessions were separated into three sex phenotypic classes: monoecious (plants with AI=1-1.35), partially andromonoecious (plants with AI=1.35-2.7), and andromonoecious (plants with AI=2.7-3). It has been reported that andromonoecy phenotype is caused by the *CitACS4* mutant allele *m* (Boualem et al., 2016; Ji et al., 2016; Manzano et al., 2016), although is also responsible for PA under heterozygous conditions. So, *MM* and *mm* plants are monoecious and andromonoecious, respectively, while *Mm* are PA, with AI ranging from 1.35 to 2.7 (Manzano et al., 2016).

During this study, we discovered that the partial andromonoecy of differing watermelon accessions, including that of P84, is not solely dependent upon the action of the *CitACS4* gene, but also upon that of a different recessive gene. We came to this conclusion in the following way: First, a number of accessions exhibited a PA phenotype, but all were homozygous for the WT allele of *CitACS4* (*MM*). Second, no variation was found in the coding sequence of monoecious and PA *MM* plants. Third, no genetic linkage was found between PA and *CitACS4* in a segregating F2 population derived from the cross P86 (*MM*, monoecious) X P84 (*MM*, PA) lines. The phenotypic segregations in the F2 (3:1 for M:PA), F3, and F4 generations (**Table 2**) confirmed that the PA phenotype is conferred by a single recessive locus called *pa*. Therefore, the three most important sex morphotypes of watermelon can be conferred by the combination of the loci *M/m* and *Pa/pa*: i) monoecious, *MM Pa_*; ii) partial andromonoecious, *MM papa* and *Mm* _ _; and iii) andromonoecious, *mm _ _*. It is possible that the *pa* locus could be equivalent to the trimonoecious (*tm*) locus proposed by Rosa (1928) and Ji et al. (2015), but these authors considered trimonoecious to the plants having the three flower types, without defining the level of andromonoecy that we calculated in this paper on the basis of the Andromonoecy Index (AI). For this reason, it is probable that the trimonoecious trait is not exactly what we have called here partial andromonoecy (PA).

An evolutionary analysis of the trait in other species of the genus *Citrullus* other than *C. lanatus* will be decisive in identifying new allelic variants in the *pa* locus and the *CitACS4* gene, and to establish their association with the level of andromonoecy. Boualem et al. (2016) found that the *CitACS4* variant responsible for andromonoecy in *C. amarus* was different from that in *C. lanatus*, but they did not report differences between the level of andromonoecy of both species. Whether other *CitACS4* variants may be linked to partial andromonoecy in *Citrullus* species requires further study. The high nucleotide diversity found in *Citrullus* species, especially in *C. colocynthis* and *C. amarus* (Guo et al., 2019), could also help not only to better define the *pa* locus found on chromosome 1, but also to identify new loci associated with partial andromonoecy in watermelon.

### Location of the *pa* Locus on a 867 kb Genomic Region of Chromosome 1

BSA-seq (Takagi et al., 2013) is a helpful alternative to the conventional gene mapping approach. It has been very successfully used for mapping several agronomic traits in differing plant species, including cucurbit species such as cucumber and melon (Pujol et al., 2019; Zhang T. et al., 2019), and watermelon (Dou et al., 2018a; Dou et al., 2018b; Guo et al., 2019; Oren et al., 2019; Zhu et al., 2019; Gebremeskel et al., 2020). Utilising an F2 segregating population and a BSA-seq approach, we were also able to quickly map the locus *pa* on a single genomic region of chromosome 1. The same region was also revealed by Genome Wide association analysis (GWAS) in different alternative panels of 122, 96 and 47 accessions of dessert watermelon. GWAS easily detected several SNPs on chromosome 3, which are tightly linked to major andromonoecious gene *CitACS4* (Boualem et al., 2016; Ji et al., 2016; Manzano et al., 2016). It also identified two SNPs on chromosome 1 as being responsible for the PA phenotype. GWAS was used to map other important agronomic traits in watermelon, melon and cucumber (Nimmakayala et al., 2016; Yagcioglu et al., 2016; Dou et al., 2018a; Dou et al., 2018b; Hou et al., 2018; Wang et al., 2018; Bo et al., 2019b; Bo et al., 2019a; Oren et al., 2019). The major sex-determining gene for monoecy/andromonoecy in melon (*CmACS7*) was also successfully detected using the GWAS approach (Gur et al., 2017; Zhang H. et al., 2019), but so far no other andromonoecy locus had been found in cucurbits. The use of panels with a greater number of watermelon accessions will surely increase the powerful of GWAS for finding the genetic variation associated with this trait.

Fine mapping narrowed down the *pa* locus to a genomic region of 867 kb containing 101 annotated genes. The region was firstly scanned for ethylene related genes, since this hormone is the main regulator of sex determination in watermelon and other cucurbits (Manzano et al., 2014; Ji et al., 2015; Manzano et al., 2016). The genomic interval has no ethylene biosynthesis or response genes but contains Chitinase- and F-box-like genes that could modulate ethylene biosynthesis and signaling pathways (**Table 4**). Arabidopsis *CHITINASE LIKE1* (*CTL1*) controls root development in etiolated seedlings by negatively regulating ethylene biosynthesis genes in response to perturbed cell wall integrity (Zhong et al., 2002; Hermans et al., 2010; Gu et al., 2019), and mutations in *CTL1* and other *Chitinase*-*like* genes overproduce ethylene and enhance responsiveness to ethylene (Gu et al., 2019). It has long been proposed that Chitinases enhance a plant’s defense against pathogens by hydrolyzing chitin, which is the main component of many fungal cell walls. However, there is increasing evidence demonstrating the role of these proteins in plant development (Grover, 2012). In tobacco, certain Chitinases that accumulate in normal stamens were found to be absent in a cytoplasmic male sterile mutant with reduced and malformed stamens (Lotan et al., 1989). Given that the Chitinase-like ClCG01G020800 watermelon gene in the PA line (P84) and the PA-bulk contain a frameshift deletion of 38 bp, it is possible to speculate that this gene could be responsible of the PA phenotype.

On the other hand, F-box proteins are important components of the ubiquitinin proteosome pathway, a regulatory system of many plant hormone receptors and signaling proteins (Yu et al., 2007), including the ethylene signaling factor EIN2 and the ethylene transcriptional activator EIN3 (Guo and Ecker, 2003; Potuschak et al., 2003; Gagne et al., 2004; Qiao et al., 2009). Moreover, the mutation *dwarf and deformed flowers 1-1* (*ddf1-1*) of rice, which alters the identity of whorls 2 an 3 floral organs, is affected in a gene that encodes for an F-box protein regulating B-class homeotic genes (Duan et al., 2012). The auxin receptor F-box protein TRANSPORT INHIBITOR RESPONSE 1 (TIR1) has recently been reported to be associated with pistil development in the unisexual flowers of bitter gourd *Momordica charantia* (Lin et al., 2016), which encourages the role of this F-box protein in sex determination within the Cucurbitaceae family.

Other flower developmental genes that map in the *pa* locus, but are not related with ethylene (**Table 4**), include: a Wuschel-related homeobox (WOX) transcription factor, which has been associated with lateral organ outgrowth and floral organ fusion in different plant species (Matsumoto and Okada, 2001; Yu et al., 2003; Vandenbussche et al., 2009); a glutaredoxin family protein, some of its members are required for stamen development in Arabidopsis (Xing and Zachgo, 2008; Reichheld et al., 2010); a GATA-like transcription factor, GATA being a protein family that plays a vital role in the functioning of various physiological and developmental processes including the development and the identity of floral organs (Zhao et al., 2004; Mara and Irish, 2008); a lateral root primordium protein, from a gene family that includes SHY/STY transcription factors involved in the promotion of the development of carpels and stamens, among other organs (Kuusk et al., 2006; Estornell et al., 2018); an FG-GAP protein and a male sterility MS5 protein, some of whose members are known to participate in anther and pollen development (Sanders et al., 1999; Zou et al., 2020).

Breeding for monoecy in watermelon will require both the selection of the monoecious *M* allele of the major gene *CitACS4* and the counter-selection of the *pa* allele associated with partial andromonoecy. Identifying the gene involved in PA will benefit the stability of monoecy in watermelon lines and commercial hybrids, but will also improve the breeding programs of other crops such as melon, cucumber and zucchini, where monoecy instability also leads to a decrease in fruit production and quality (Boualem et al., 2009; Díaz et al., 2014; Martínez et al., 2014; Tan et al., 2015; Manzano et al., 2016; Martos-Fuentes et al., 2017; Aguado et al., 2018).

## Data Availability Statement

The datasets generated for this study were deposited to European Variation Archive (EVA: https://www.ebi.ac.uk/eva) with the project accession number PRJEB39381.

## Author Contributions

EA conducted most of the experiments. AG collaborated in the phenotyping of the accessions, and developed and evaluated breeding material. JI-M and JR collaborated in the phenotyping the accessions. TW collaborated on field phenotyping of USDA accessions. BP, MG-G, and AG-C identified and selected the studied Spanish accessions. EA, CM, and MJ performed data and mapping analysis, and wrote the manuscript. MJ and CM designed and coordinated the research.

## Funding

This work has been funded by grant UAL18-BIO-B017-B, awarded by the call “Proyectos UAL-FEDER “ within the framework of the 2014-2020 FEDER-Andalusia Operational Program, as well as by the research group BIO293 of the University of Almería.

## Conflict of Interest

The authors declare that the research was conducted in the absence of any commercial or financial relationships that could be construed as a potential conflict of interest.

The reviewer UR declared a past co-authorship with one of the authors BP to the handling editor.
