# Supplementary material for: Mapping a Partial Andromonoecy Locus in Citrullus lanatus Using BSA-Seq and GWAS Approaches
Source: Front Plant Sci. 2020 Aug 19;11:1243. doi: 10.3389/fpls.2020.01243 (PMC7466658; doi:10.3389/fpls.2020.01243)
Supplement: Supplementary file 1 [file Presentation_1.zip › Supplementary Figure 1.pptx]

## Slide 1
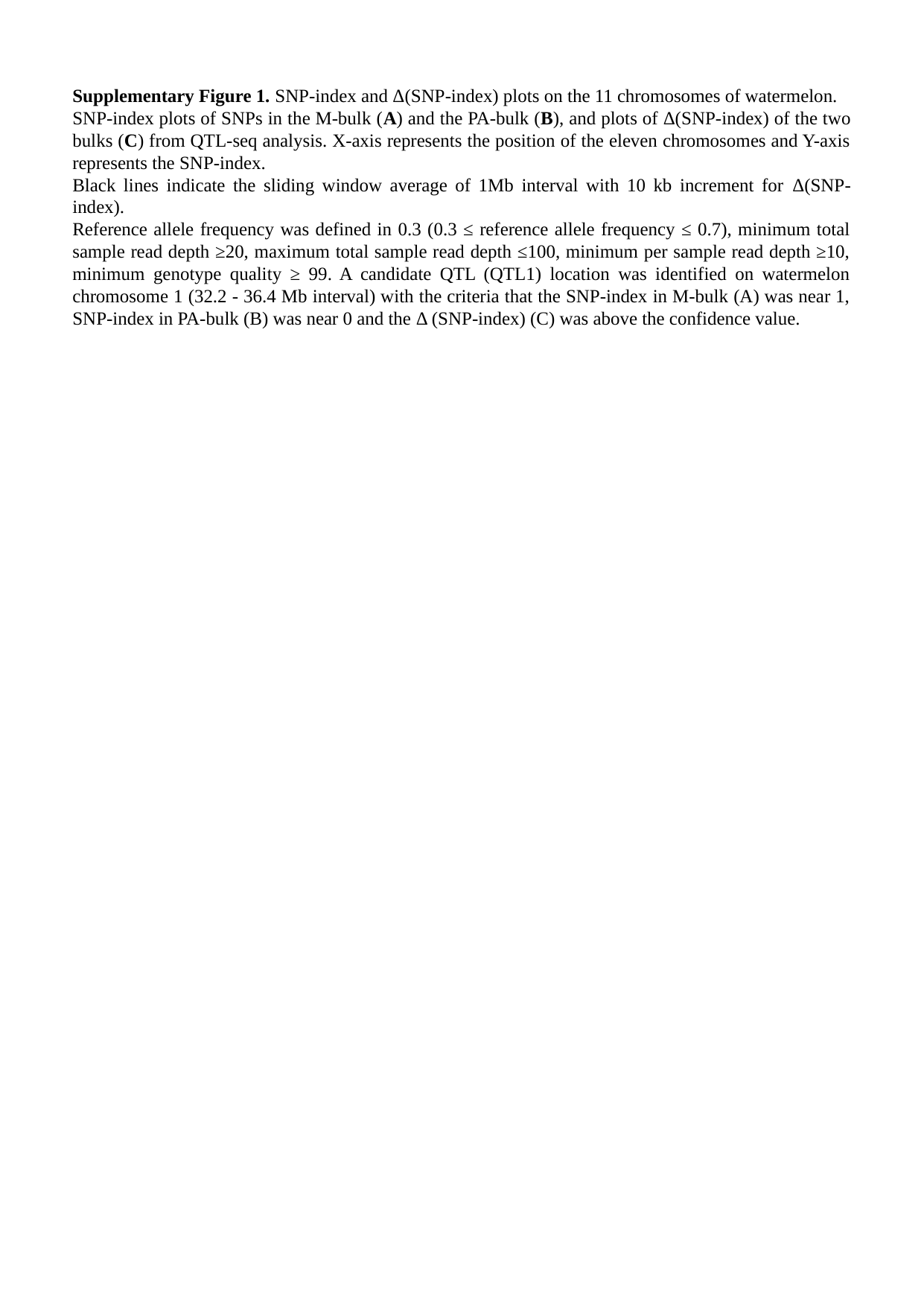

Supplementary Figure 1. SNP-index and Δ(SNP-index) plots on the 11 chromosomes of watermelon.
SNP-index plots of SNPs in the M-bulk (A) and the PA-bulk (B), and plots of Δ(SNP-index) of the two bulks (C) from QTL-seq analysis. X-axis represents the position of the eleven chromosomes and Y-axis represents the SNP-index.
Black lines indicate the sliding window average of 1Mb interval with 10 kb increment for Δ(SNP-index).
Reference allele frequency was defined in 0.3 (0.3 ≤ reference allele frequency ≤ 0.7), minimum total sample read depth ≥20, maximum total sample read depth ≤100, minimum per sample read depth ≥10, minimum genotype quality ≥ 99. A candidate QTL (QTL1) location was identified on watermelon chromosome 1 (32.2 - 36.4 Mb interval) with the criteria that the SNP-index in M-bulk (A) was near 1, SNP-index in PA-bulk (B) was near 0 and the Δ (SNP-index) (C) was above the confidence value.

## Slide 2
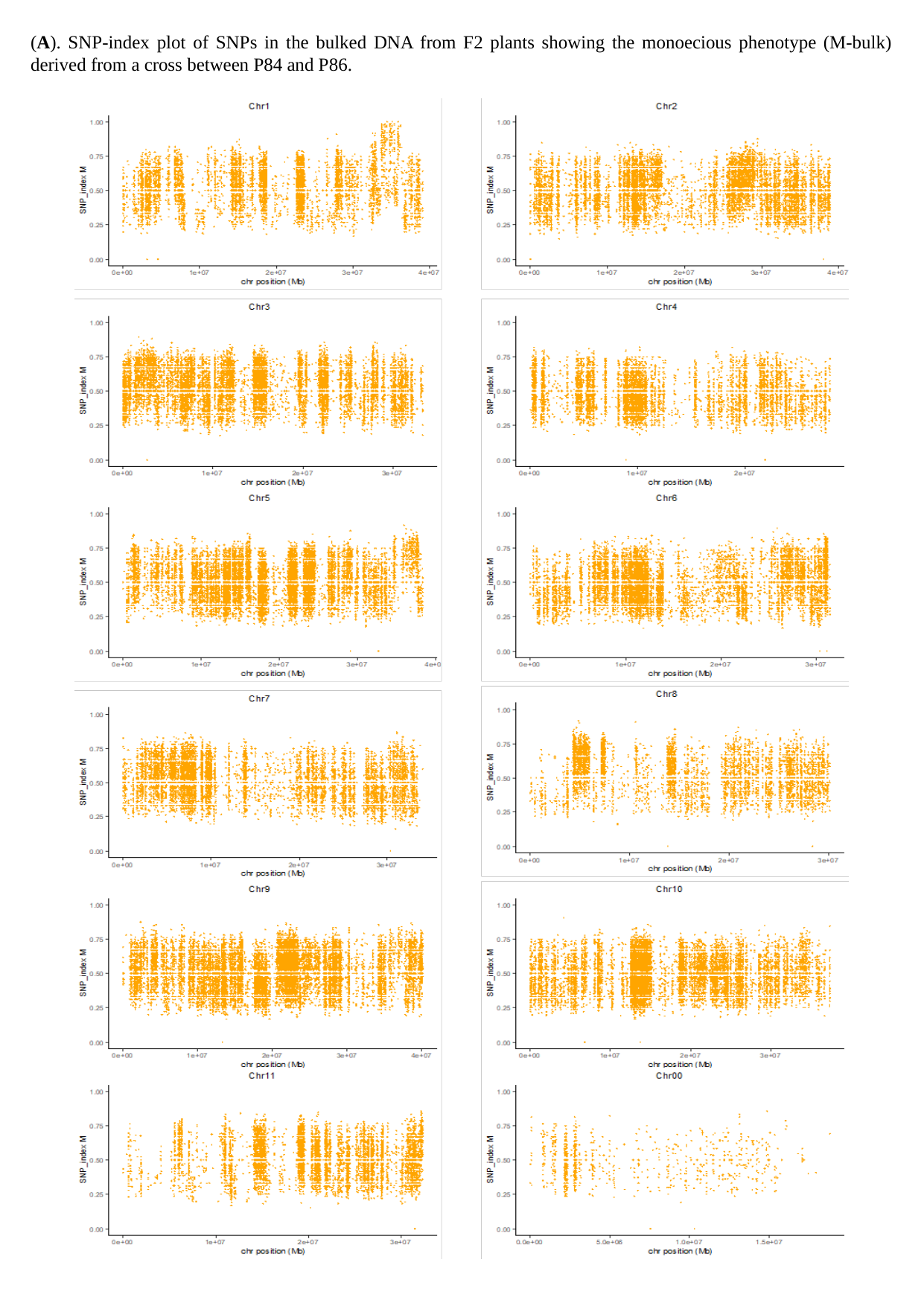

(A). SNP-index plot of SNPs in the bulked DNA from F2 plants showing the monoecious phenotype (M-bulk) derived from a cross between P84 and P86.

## Slide 3
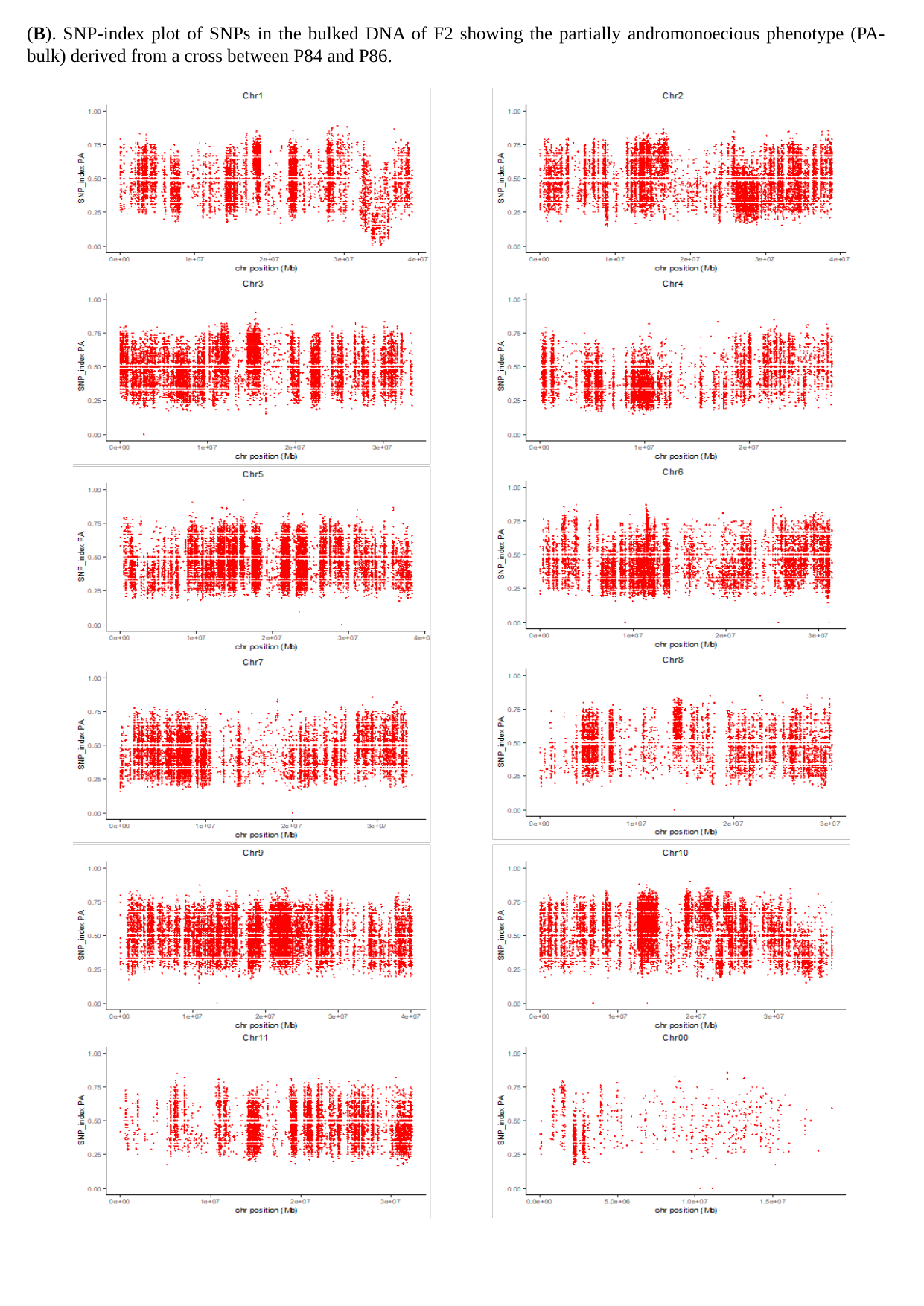

(B). SNP-index plot of SNPs in the bulked DNA of F2 showing the partially andromonoecious phenotype (PA-bulk) derived from a cross between P84 and P86.

## Slide 4
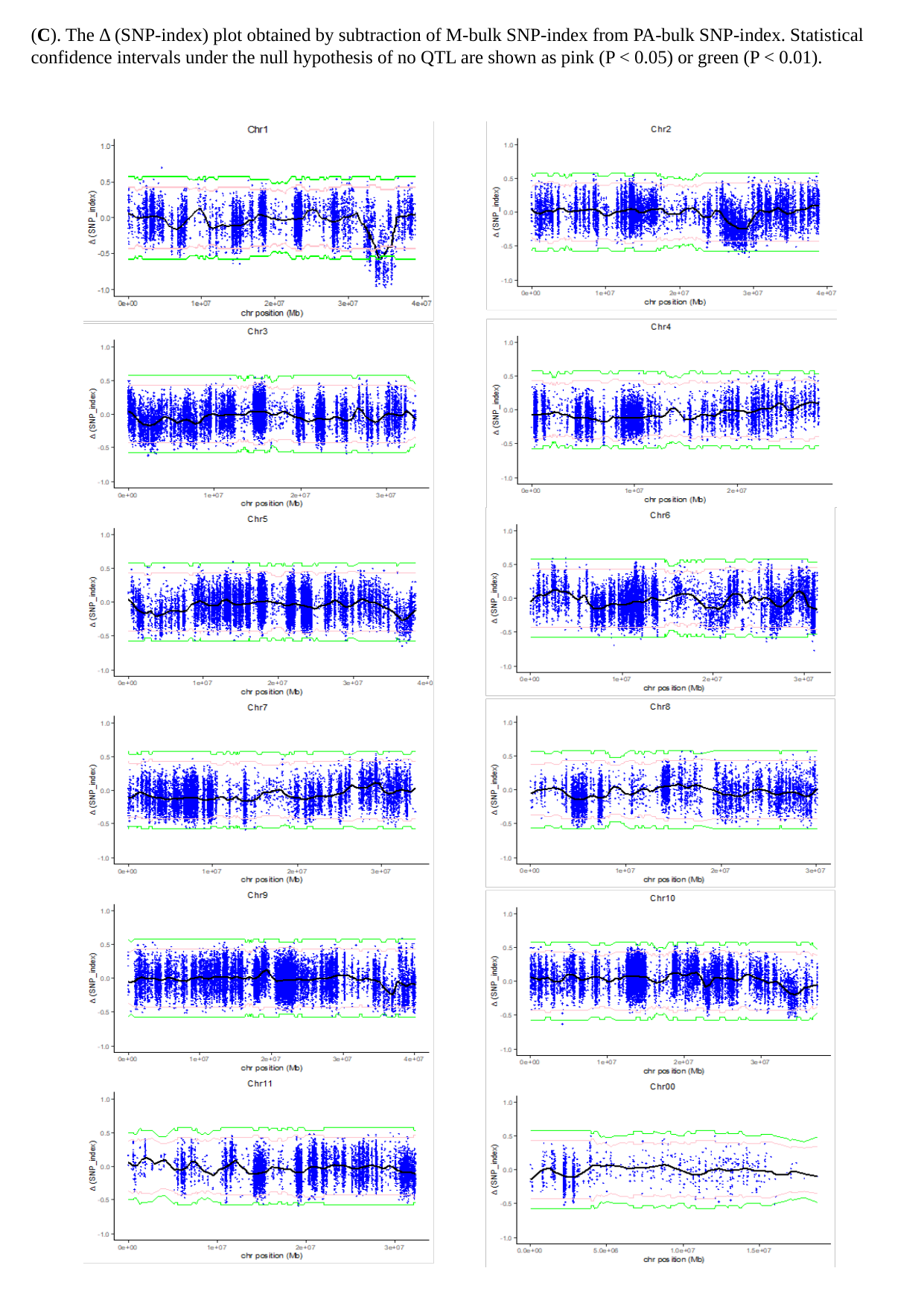

(C). The Δ (SNP-index) plot obtained by subtraction of M-bulk SNP-index from PA-bulk SNP-index. Statistical confidence intervals under the null hypothesis of no QTL are shown as pink (P < 0.05) or green (P < 0.01).
