# Supplementary figures and images for: Mapping a Partial Andromonoecy Locus in Citrullus lanatus Using BSA-Seq and GWAS Approaches
Source: Front Plant Sci. 2020 Aug 19;11:1243. doi: 10.3389/fpls.2020.01243 (PMC7466658; doi:10.3389/fpls.2020.01243)

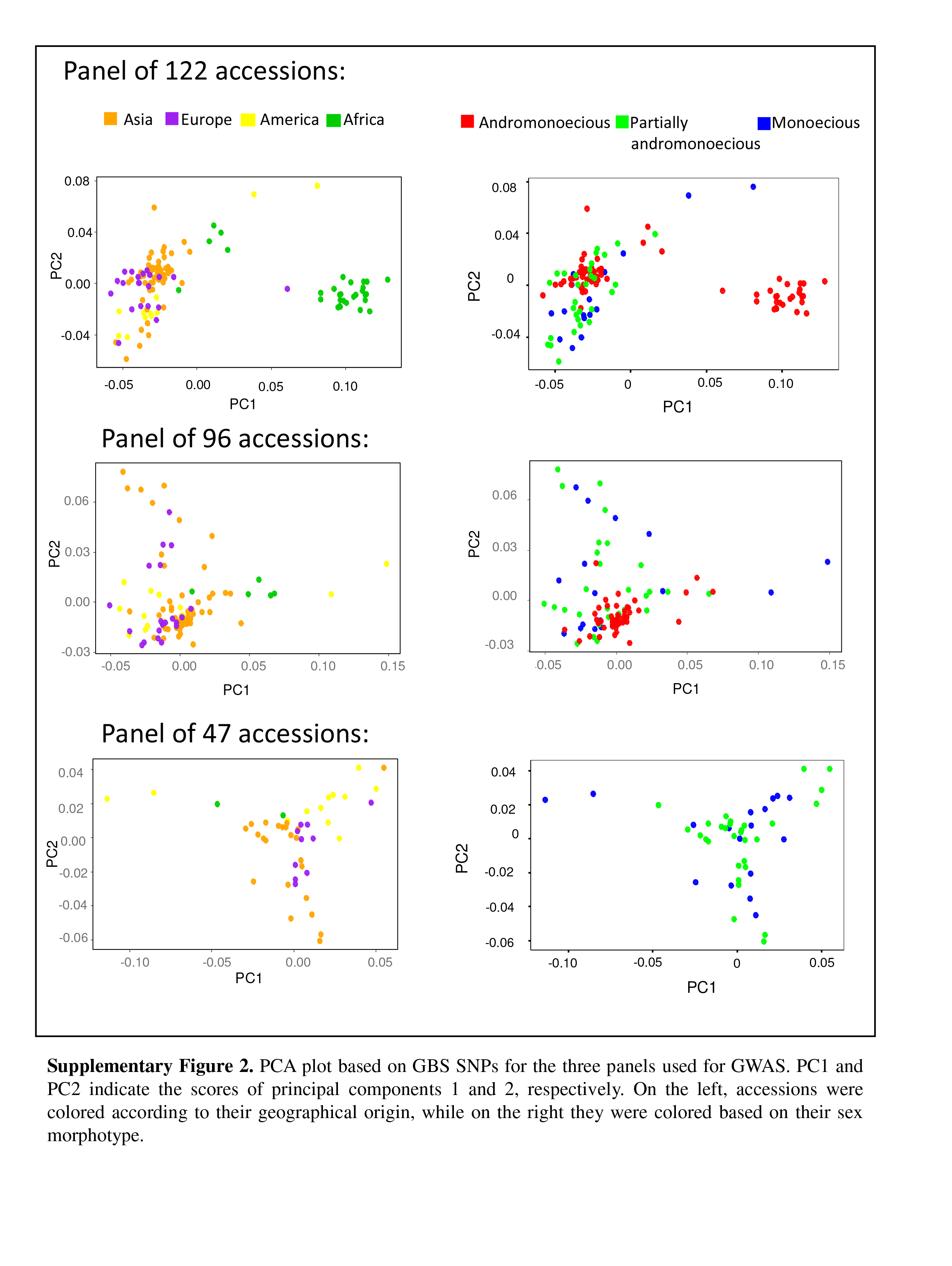

Supplement: Supplementary file 1 [file Presentation_1.zip › Supplementary Figure 2.tif]
